# Two genetic variants explain the association of European ancestry with multiple sclerosis risk in African-Americans

**DOI:** 10.1038/s41598-020-74035-7

**Published:** 2020-10-09

**Authors:** Nathan Nakatsuka, Nick Patterson, Nikolaos A. Patsopoulos, Nicolas Altemose, Arti Tandon, Ashley H. Beecham, Jacob L. McCauley, Noriko Isobe, Stephen Hauser, Philip L. De Jager, David A. Hafler, Jorge R. Oksenberg, David Reich

**Affiliations:** 1 Department of Genetics, Harvard Medical School, New Research Building, Boston, MA 02115 USA; 2 Harvard-MIT Division of Health Sciences and Technology, Harvard Medical School, Boston, MA 02115 USA; 3 Department of Human Evolutionary Biology, Harvard University, 16 Divinity Ave., Cambridge, MA 02138 USA; 4 Broad Institute of Harvard and Massachusetts Institute of Technology, Cambridge, MA 02141 USA; 5 Systems Biology and Computer Science Program, Department of Neurology, Ann Romney Center for Neurological Diseases, Brigham & Women’s Hospital, Boston, MA 02115 USA; 6 Division of Genetics, Department of Medicine, Brigham & Women’s Hospital, Harvard Medical School, Boston, MA 02115 USA; 7 Department of Bioengineering, University of California Berkeley, San Francisco, Berkeley, CA 94720 USA; 8 John P. Hussman Institute for Human Genomics, Miller School of Medicine, University of Miami, Miami, FL 33136 USA; 9 Dr. John T. Macdonald Foundation Department of Human Genetics, Miller School of Medicine, University of Miami, Miami, FL 33136 USA; 10 Department of Neurology, University of California San Francisco School of Medicine, San Francisco, CA 94158 USA; 11 Present Address: Department of Neurology, Graduate School of Medical Sciences, Kyushu University, Fukuoka, Fukuoka 812-8582 Japan; 12 Department of Neurology, Center for Translational & Computational Neuroimmunology, Columbia University Irving Medical Center, New York, NY 10032 USA; 13 Departments of Neurology and Immunobiology, Yale School of Medicine, New Haven, CT 06520 USA; 14 Howard Hughes Medical Institute, Harvard Medical School, Boston, MA 02115 USA

**Keywords:** Multiple sclerosis, Medical genomics

## Abstract

Epidemiological studies have suggested differences in the rate of multiple sclerosis (MS) in individuals of European ancestry compared to African ancestry, motivating genetic scans to identify variants that could contribute to such patterns. In a whole-genome scan in 899 African-American cases and 1155 African-American controls, we confirm that African-Americans who inherit segments of the genome of European ancestry at a chromosome 1 locus are at increased risk for MS [logarithm of odds (LOD) = 9.8], although the signal weakens when adding an additional 406 cases, reflecting heterogeneity in the two sets of cases [logarithm of odds (LOD) = 2.7]. The association in the 899 individuals can be fully explained by two variants previously associated with MS in European ancestry individuals. These variants tag a MS susceptibility haplotype associated with decreased *CD58* gene expression (odds ratio of 1.37; frequency of 84% in Europeans and 22% in West Africans for the tagging variant) as well as another haplotype near the *FCRL3* gene (odds ratio of 1.07; frequency of 49% in Europeans and 8% in West Africans). Controlling for all other genetic and environmental factors, the two variants predict a 1.44-fold higher rate of MS in European-Americans compared to African-Americans.

## Introduction

Admixed populations are formed when two populations with divergent ancestries have offspring. Admixture mapping is a method to screen through the genome, searching for loci where individuals with a disease from an admixed population tend to have a significantly different proportion of one ancestry than their population’s genome-wide average. For example, admixture mapping in African-Americans involves searching genomes for areas where European ancestry deviates from the average, which is roughly 20% in African-Americans albeit with substantial variation across the United States^1^. These genomic loci of high deviation in local ancestry indicate the presence of disease risk variants that differ in frequency between the ancestral populations, which can then be followed up using fine-mapping approaches. To date, admixture mapping has successfully identified genomic loci that were then fine-mapped to identify genetic risk variants for several diseases, notably prostate cancer and end-stage renal disease^2–5^.

The first successful admixture mapping study was for multiple sclerosis (MS) in African-Americans, a good candidate disease for this method because early prevalence studies suggested that individuals of European ancestry have significantly higher rates of MS than African-Americans (1.49- to 2.27-fold)^6–8^. However, more recent studies have disputed the epidemiological observation, with one showing African-American women having a 1.59-fold higher risk than European-American women and African-American men having the same risk as European-American men^9^. Another study suggested that African-Americans of both sexes have a 1.27-fold higher risk of MS than European-Americans^10^. In 2005, admixture mapping analysis led to the discovery of a genetic risk factor for MS near the centromere of chromosome 1 estimated to lead to an approximately 1.44-fold increase in MS risk per allele of European ancestry relative to African ancestry^11^. Since the publication of that study, joint analyses of genome-wide association studies (GWAS) and targeted SNP genotyping, largely focused on people of European ancestry, have discovered 201 genetic risk variants for MS outside of the MHC (Major Histocompatibility Complex) region and 32 variants within the MHC region^12–17^. However, there is evidence for incomplete overlap between African-American and European MS risk variants^18^, and the specific variants responsible for the 2005 admixture association in African-Americans have to date been unresolved.

We analyzed 1305 African-American MS cases and 1155 African-American controls and found that the 2005 admixture mapping signal could be fully explained by two variants that are strongly correlated with haplotypes previously linked to MS in people of European ancestry: one in the *CD58* gene located on the p-arm near the centromere and one in the *FCRL3* gene on the q-arm near the centromere. We suggest that the localization of the admixture association signal over the centromere of chromosome 1 is due to a combined association of risk factors straddling the centromere.

## Results

### Replication of chromosome 1 signal

As part of a large replication study of multiple sclerosis risk variants, a custom genotyping array (MS Chip) was designed using the Illumina platform that included 321,105 single nucleotide polymorphisms (SNPs) across the genome that were genotyped in 24,770 cases with multiple sclerosis (of which 1305 were African-American) and 23,193 controls without multiple sclerosis (of which 1155 were African-American)^15^. A total of 9014 SNPs were incorporated into the array specifically to study the chromosome 1 admixture mapping result published in 2005, including 4014 Ancestry Informative Markers (AIMs, known to be highly differentiated between people of West-African and European ancestry) spread genome-wide, and 5000 SNPs within the chromosome 1 admixture mapping peak, which we designed to provide dense coverage in both European and African haplotype backgrounds and to include SNPs in non-repetitive regions within the chromosome 1 centromere. Detailed design specifications of the SNP array are given elsewhere^15^.

We initially analyzed all 1305 African-American MS cases and 1155 African-American controls together (see Supplementary Table S1 for sample details) using the ANCESTRYMAP software^1^, which calculates LOD scores across the genome as a Bayesian likelihood ratio of the genetic site’s likelihood of the data under the specified disease model divided by the likelihood of the data under no disease model. We found a signal near the centromere of chromosome 1 (maximum LOD score of 2.7) that was significantly weaker than the result of the 2005 scan that had obtained a score of 5.2 in 605 cases^11^ (which met the published threshold for genome-wide significance of 5) (Table 1, Supplementary Fig. S1). We were perplexed by this result, as the maximum LOD score at chromosome 1 had increased to 9.3 in a follow-up study in 2007 where the sample size was increased to 1044 cases albeit using a less dense set of AIMs than in the study reported here and using a mixture of genotyping methods^19^. When we restricted the analysis of our new data to the 899 MS subjects that overlapped the 2007 study, the maximum LOD on chromosome 1 went up to 9.8 (Supplementary Fig. S2) in a region overlapping the centromere (physical position 116–164 Mb in hg19 genomic coordinates). In addition, when we used a permutation test to determine an empirical p-value, this was significant at P < 0.001 (Table 1). This provided a technical validation of the 2007 result using a different genotyping platform on the same samples.

**Table 1:** Admixture association scores on chromosome 1 in different sample sets.

Study	Cases	Controls	Highest LOD score on chr. 1 case-only	Highest LOD score on chr. 1 cases + controls	Highest case–control Z-score on chr. 1	Empirical p-value for admixture association	95% CI for risk per European chromosome
2005 study	605	1043	5.2	5.2	3.3	N/A	1.27–1.70
2007 follow-up	1044	1161	9.2	9.3	4.2	< 0.001	1.32–1.62
Full new cohort	1305	1155	3.9	2.7	3.4	0.114	1.16–1.43
2007 subset of new cohort	899	1155	9.8	9.3	4.5	< 0.001	1.37–1.76
Samples added after 2007	406	1155	− 3.6	− 3.0	0.9	1	0.59–0.91

The highest LOD score results are computed by ANCESTRYMAP based on a prior on relative risk per European ancestry allele of 1.5. The 95% confidence intervals for risk per European allele are obtained by running on a uniformly spaced grid of models from 0.5 to 2.0-fold per European allele, assuming an equal prior probability of risk for each, and then taking the LOD score to the power of 10 and normalizing to obtain a posterior. The LOD scores for the grid of models for the 2005 study are from the original publication^11^; all LOD scores are given in Supplementary Table S3. Empirical p-values were found through permutation analysis; see “Materials and methods” section. p-values were listed as < 0.001 when 0 of the 1000 permutations had a score at least as large as that of the LOD score of that data subset.

We separately analyzed the 406 new MS cases that did not overlap with the 2007 cases and found no significant signal at the chromosome 1 locus. Indeed, the 95% confidence interval for risk for MS per copy of European ancestry is significantly below that of African ancestry for the post-2007 cases (0.59–0.91-fold per copy of European ancestry), compared to a non-overlapping 95% confidence interval of 1.37–1.76-fold for the 2007 cases. This suggests an opposite effect of increased risk due to African ancestry at this locus, not increased risk due to European ancestry (Table 1, Supplementary Fig. S3). Comparing the cases with data available to us in 2007 and the 406 post-2007 cases, we could not detect any difference in genome-wide ancestry proportions, sex proportions (ratio of females to males), quality control measures (genotyping error rate or PCA clustering, including after restricting to the region around the centromere of chromosome 1) (Supplementary Fig. S4), origin of DNA (cell line vs. genomic DNA or location of sample collection), or Native American ancestral contribution (Supplementary Table S1). Study inclusion/exclusion criteria remained the same and were strictly implemented for all datasets. When we removed all samples known to be of Afro-Caribbean origin, the results did not change significantly. When we analyzed the HLA-DRB1 status (known to be associated strongly with MS^20^), the 2007 cases and the new cases did not show any significant difference in association to MS (Supplementary Table S1). The cases genotyped after 2007 had a lower average copy number for the HLA-DRB1*15:01/15:03 haplogroup (38%) relative to the cases available as of 2007 (42%), which meant that the elevation of the frequency of this haplogroup relative to the controls (34%) was significant for the cases available as of 2007 but not for the ones added afterward. Along with the attenuation of the admixture mapping signal, this result suggests that the cases collected up until 2007 may have been more enriched for people with genetic susceptibility to MS than the controls. We have no evidence that a sample mix-up occurred, and overall we could not find any significant difference between the 2007 cases and the new cases beyond the inhomogeneity in their contribution to the chromosome 1 admixture mapping peak. Nevertheless, motivated by the validation of the admixture signal in 2007 and access to high-density new genotyping data, we proceeded to use these data to understand the basis of this signal, restricting to the 899 cases that overlapped between the 2007 and 2016 studies as it was in this subset of cases that we had a strong ancestry association that we could parse in terms of specific variants contributing to it.

### Determining the basis of the chromosome 1 signal

**Figure 1:**
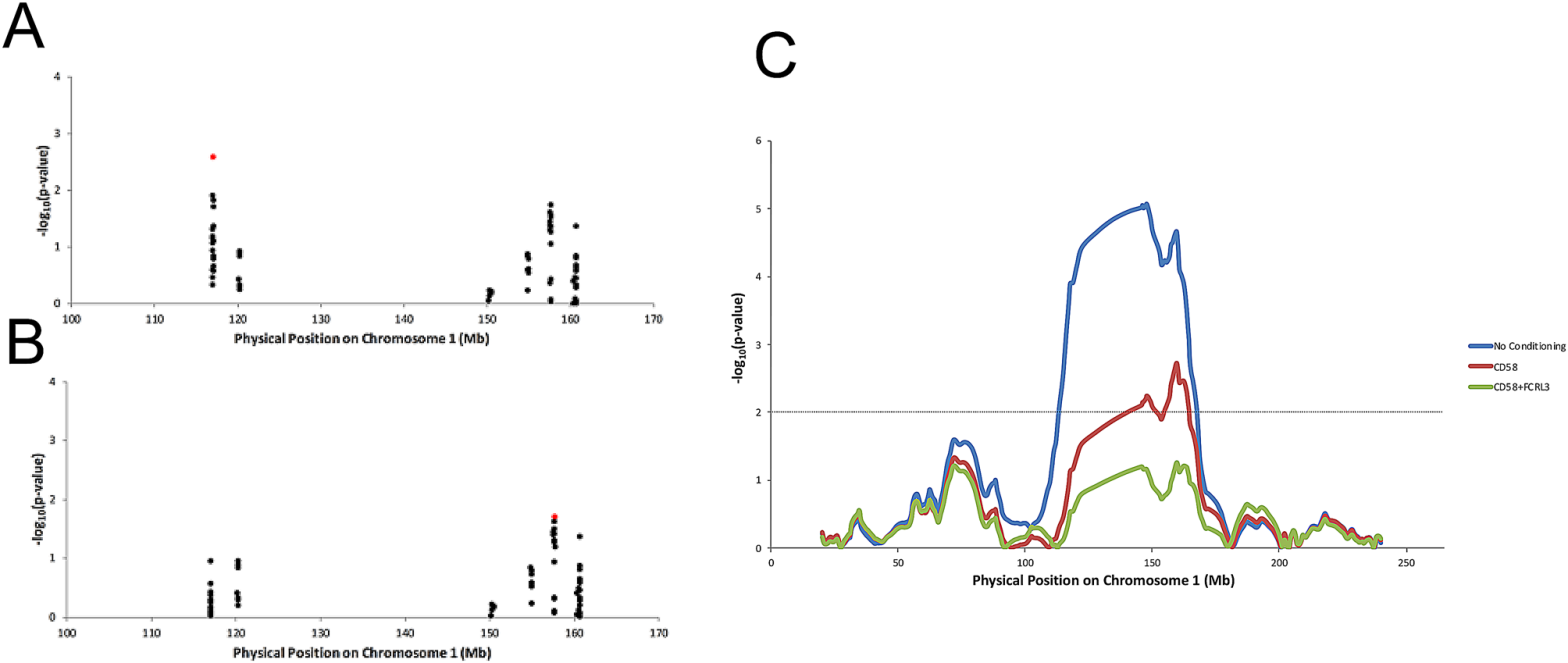
Two variants are sufficient to explain the admixture association signal. (**A**,**B**) Top GWAS variants in the region of the admixture association signal (red box) were taken and used in a logistic regression for genotype association on MS case–control status in African-American data after conditioning on global and local European ancestry. Y-axis is − log_10_ (p-value) of association with MS case status. Shown in red are the most highly associated variants, rs12025416 in the *CD58* gene for (**A**) and rs6681271 in the *FCRL3* gene in (**B**) (after conditioning on the top variant in **A**). (**C**) Logistic regression of local European ancestry on case–control status in African-American data after controlling for global ancestry as a covariate as well as the top variants from (**A**,**B**). No conditioning indicates only controlling for global ancestry. The dotted line indicates threshold for significance (this p-value threshold represents a lower bound on significance due to the fact that the peak can shift after conditioning). The African-American data used for all analyses was the 2007 subset of the new cohort.

We searched for potential causal variants in the chromosome 1 admixture mapping peak, which we defined for this analysis as 114–162 Mb on chromosome 1 in hg19 genome coordinates (that is, the area with LOD score > 5 in the admixture analysis adding 2 Mb on either side; Fig. 1). The African-American sample size was insufficient to obtain any genome-wide significant associations (Supplementary Fig. S5) as suggested by power estimates (Supplementary Table S1). We thus began by narrowing our search to variants with some evidence for association with MS from a study of a much larger sample size of people of European ancestry who were genotyped on a custom SNP array designed to test for association to MS^15^ (Supplementary Table S2, Supplementary Fig. S6), making the plausible assumption that the variants associated with disease risk in European-Americans are also risk factors in African-Americans^18^. Seventy-nine variants in the region had a p-value < 10^–5^ in the European ancestry association study, and we focused our analysis on these 79 variants in all subsequent analyses. Within the admixture mapping peak, the European ancestry association study had identified seven independent genetic tag variants that together were sufficient to account for all the genome-wide significant association to MS detected in that study (that is, controlling for the allelic status of these variants, there was no additional genome-wide evidence of association to MS in the genomic region)^15^. However, we found that these seven tag SNPs were not sufficient to account for the association to MS detected in African-Americans, as if we condition on the allelic status of all seven following the procedure discussed in what follows, there is still a residual ancestry association signal in African-Americans with LOD = 3.6.

In light of the fact that the tag SNPs from the European genetic map were insufficient to explain the admixture association signal in African-Americans, we turned to studying the 79 variants significant at p < 10^−5^ in the European ancestry association study and evaluating their contribution to MS in African-Americans. Specifically, we used these variants to perform a logistic regression in the African-American data of SNP genotype association on case–control status while controlling for genome-wide and local European ancestry as covariates (Fig. 1, Supplementary Table S3). Because we controlled for ancestry in this analysis, this procedure treated the SNP association signals completely independently from the ancestry association signals, allowing us to drive the analysis entirely by SNP association results and only afterward to determine whether the chosen SNPs explained the admixture mapping peak.

The SNP with the strongest association to MS in the African-American data was rs12025416 in the vicinity of the *CD58* gene. After using this SNP as a covariate in a logistic regression of European ancestry on case–control status with global ancestry also as a covariate, the significant ancestry association signal disappeared on the p-arm (p > 0.01 for the ancestry association and p > 0.05 for the genotype associations at all SNPs). However, an ancestry association signal on the q-arm remained (p < 0.01). When we instead used the *CD58* SNP with the strongest signal in the European MS Chip association analysis (rs1335532)^15^, the ancestry association signal on the p-arm was still present (p < 0.005), confirming the value of guiding our analysis using the SNP associations measured directly in African-Americans; that is, on focusing on the most strongly associated SNP from the African-American scan rather than the most strongly associated SNP from the European scan (Supplementary Table S3). In the ancestry association tests controlling for SNP association here and in what follows, we also carried out follow-up analyses re-inferring ancestry after masking all positions within 1 centi-Morgan (cM) of the variants whose SNP associations we were using as covariates to address the potential pitfall that SNPs used in the admixture analysis might be in tight LD with these SNPs. These sub-analyses did not lead to any significant change in our results.

We next took the most highly associated variant after conditioning on allelic status of rs12025416, which was rs6681271 in the vicinity of the *FCRL3* gene (Fig. 1). After using rs6681271 jointly with rs12025416 as covariates, there was no residual association in African-Americans across the entire admixture mapping peak (p > 0.05 for both ancestry association and genotype association)*.* When any significant variants (from the 79 above) on the q-arm were used as covariates instead of rs6681271, only one, rs7528684 also in the *FCRL3* gene, produced more of a decrease in the ancestry association signal. However, we cannot statistically distinguish between the signals and hence we have chosen to tag the *FCRL3* association using rs6681271, which we obtained through a formal procedure of using SNP associations entirely independently of ancestry associations (Supplementary Table S3). Similar to the *CD58* variant, when we instead conditioned on the strongest associated *FCRL3* variant from the European MS Chip association analysis (rs3761959)^15^, some ancestry association signal on the q-arm remained (p < 0.005) (Supplementary Table S3), reaffirming how the European MS Chip association cannot by itself identify variants that explain the signal of admixture association in African-Americans. Thus, the African-American data adds additional valuable information about MS risk even with its much smaller sample size.

After controlling for the top two variants associated to MS risk in African-Americans, there is no significant MS risk at *CD58* (residual association of p = 0.50 at the European study tag SNP rs1335532) or *FCRL3* (residual association of p = 0.22 at the European study tag SNP rs3761959). In contrast, as discussed above, controlling for those top two variants from the European study and indeed all seven across the MS peak does not account for all evidence of association in African-Americans (after conditioning on all seven SNPs, there is a residual SNP association at rs12025416 in *CD58* of p = 0.039 and at rs6681271 in *FCRL3* of p = 0.0082; the residual admixture association has LOD = 3.6).

Why is it that the seven variants that were the best tag SNPs in the region from the European genetic map cannot explain the admixture association in African-Americans, whereas the top two SNPs identified in an African-American association scan are able to explain it? A possible explanation is that shorter average linkage disequilibrium (LD) in genomic segments of African ancestry compared to European ancestry allow us to more finely map the true causal variants, which by implication would be different from the tag SNPs that emerged from the European ancestry association study. The variants we identify as associated to MS are in high LD with the SNPs in the same gene identified in the association study in people of European ancestry (r^2^ = 0.73 between rs12025416 and rs1335532, and r^2^ = 0.89 between rs6681271 and rs3761959, Supplementary Table S4; they are on the same risk haplotype) but less so in people of African ancestry (r^2^ = 0.23 between rs12025416 and rs1335532, and r^2^ = 0.20 between rs6681271 and rs3761959). While the higher frequencies of the risk alleles in people of European ancestry than of African ancestry for both tag SNPs means that the LD structure in Europeans is more relevant for these SNPs in African-Americans than might be expected from the overall proportion of European ancestry in the population, a substantial fraction of the risk alleles in African Americans are still coming from an African ancestry background given the overall very high proportion of African ancestry in African-Americans, and this decreases the relevance of the European tag SNPs.

In the 1000 Genomes Phase 3 data, the allele tagging the *CD58* risk haplotype (rs12025416) has a frequency of 83.8% in CEU (European-Americans of Northern European ancestry), 21.8% in YRI (Yoruba of Nigeria), and 31.9% in CHB (Han Chinese) and JPT (Japanese). In the European MS Chip association, it had a p-value of 3.32 × 10^–32^ and an effect size (odds ratio) of 1.37 (Table 2), one of the strongest risk alleles for MS outside of the MHC. The *FCRL3* risk variant (rs6681271) has a frequency of 49.0% in CEU, 7.9% in YRI, and 56.5% in CHB and JPT. In the European MS Chip association study, it had a p-value of 3.24 × 10^–6^ and odds ratio of 1.07. If one makes the simplifying assumption of a constant (fixed) effect size in all populations, the population attributable risk (PAR—the expected reduction in MS incidence if the risk alleles did not exist in the population) for both variants together was 45% for European-Americans, 15% for West Africans, and 21% for African-Americans. (For just the *CD58* variant, the PAR is 41% for European-Americans, 14% for West Africans, and 20% for African-Americans. For just the *FCRL3* variant the numbers are 7% for people of European ancestry, 1% for West Africans, and 2% for African-Americans.) This implies a 1.44-fold higher risk for MS in European-Americans than in African-Americans, after controlling for all other genetic and environmental factors (1.44 = (100–21%)/(100–45%)).

**Table 2:** p-values for association with MS status in different datasets.

Dataset	p-value for association with MS status at top *CD58* variant	p-value for association with MS status at top *FCRL3* variant
Europeans	3.32 × 10^−32^	3.24 × 10^−6^
African-Americans before conditioning	2.51 × 10^−3^	1.80 × 10^−2^
African-Americans after conditioning on top *CD58* variant	1.10 × 10^−1^	1.97 × 10^−2^
African-Americans after conditioning on top *CD58* and *FCRL3* variants	1.24 × 10^−1^	5.83 × 10^−2^

European dataset is the MS Chip association signal^15^. African-American dataset is the 2007 subset of the new cohort. The *CD58* variant is rs12025416, and the *FCRL3* variant is rs6681271.

Another way to assess the epidemiological effect on relative risk for MS in European-Americans compared to African-Americans is to directly use the ancestry association. Focusing on the estimated risk per copy of European ancestry in the 899 cases available up until 2007 and included in most of the analyses in this study, we infer the increased risk per copy of European ancestry to be 1.54 per copy of European ancestry at the chromosome 1 locus (95% confidence interval of 1.37–1.76; Table 1). This implies that African-Americans with two copies of European ancestry at the locus (corresponding to roughly 4% = (20%)*(20%) of African-Americans) have 1.93-fold higher risk for MS than the average African-American (95% confidence interval of 1.67–2.33). This is higher than the increased risk in European-Americans of 1.44 computed just based on the two variants (above), and suggests the possibility that their effect size estimates in European-Americans may be underestimates of those in African-Americans, perhaps reflecting different gene-environment interactions. Alternatively, using the estimates of ancestry association in the full cohort of 1305 individuals (Table 1), we obtain an estimate of 1.47-fold increased risk in African-Americans carrying two European copies at the site relative to African-Americans (95% confidence interval 1.26–1.73), which is in line with the SNP association results.

## Discussion

We sought to determine the specific genetic variants contributing to difference in risk for MS between Africans and Europeans attributable to the chromosome 1 centromere region. Using data from a custom-built genotyping platform designed specifically for studying multiple sclerosis risk including the chromosome 1 admixture association, we replicated the original admixture mapping signal on the centromere of chromosome 1 in the 899 cases that overlapped with a 2007 follow-up of the 2005 study. This demonstrates that the association found in 2005 was not likely to be due to an artifact of genotyping or a technical error in the admixture mapping software. We have not been able to provide an explanation for the post-2007 cases lacking the signal, as the signal in the post-2007 cases is significantly different from the signal in the cases available until 2007.

Nevertheless, we identified two variants that are sufficient to explain the ancestry association signal in the 899 case group, which are associated on a per-allele level to MS in a high-powered study of people of European ancestry. The variants are associated with the *CD58* and *FCRL3* genes^13^. The two variants together would be expected to lead to a 1.44-fold higher rate of MS in European-Americans compared to African-Americans, with the increase primarily driven by the *CD58* variant. The variants found in this study were not the most statistically significant effects found in the most recent genetic map of MS in Europeans^15^, but they do reside in the same haplotype in European ancestry reference populations. In African ancestry reference data, the respective LD is notably smaller, driven by allele frequency differences, suggesting that the variants identified in the European admixture scan are tags and not the true variants, and demonstrating the added value for fine-mapping provided by data from people of African ancestry.

Given the current epidemiological uncertainty and disagreement in various studies over the prevalence of MS in European-Americans compared to African-Americans, our findings cannot fully account for reported epidemiological differences in prevalence between individuals of European compared to African ancestry, and indeed it is certainly the case that there are other important genetic and environmental effects that modulate MS prevalence and incidence rates across populations and in changing environments. Nevertheless, it is remarkable that these two genetic variants by themselves, irrespective of other factors, would be sufficient to predict an increased risk in Europeans above that of Africans approximating the range that has been documented in some epidemiological studies (~ 1.49–2.27-fold). An admixture mapping study of individuals with MS^21^ that used a subset of the individuals in this study did not find any signal outside of the MHC region, except a minor one on chromosome 8 in Hispanic individuals. However, most of those individuals were genotyped without dense coverage near chromosome 1, so it is possible that this study lacked power to find the signal that we have reported here.

Additional fine-mapping of the regions identified in this study will be necessary to determine the true causal SNPs driving the association to MS. This ultimately will require experimental manipulation of candidate SNPs (after narrowing down to a smaller set of candidates in silico^22^) in relevant cell lines to determine whether they affect expression of the associated genes (or ones nearby, perhaps through affecting enhancer or transcription factor binding^23^). However, previously published experimental work has already begun to provide insight into the mechanism by which the CD58 gene variant contributes risk for MS. CD58, the protein coded for by the *CD58* gene, is a cell adhesion molecule present on antigen presenting cells (APCs) that binds to CD2 on T cells to both strengthen the adhesion between T cells and APCs and to enhance T cell activation^24^. *CD58* is also expressed in B cells, an important cell type in MS pathology, with higher levels of expression linked both to enhanced migration to inflammatory sites (for anti-inflammatory activity) and to CD2 ligation^25^. A past study showed that the protective allele of the rs2300474 variant, in strong LD with the protective allele of the rs12025416 variant, increases *CD58* levels^17^ (in contrast, the MS susceptibility haplotype, which harbors the risk allele of this variant, decreases CD58 expression). This protective effect is supported by finding that *CD58* mRNA is higher in MS subjects during clinical remission^17^. Engagement of the CD58 receptor, CD2, up-regulates the expression of transcription factor FoxP3 leading to the enhanced function of CD4^+^CD25^high^ regulatory T cells^17^ that are defective in subjects with MS^26^. In this regard, a CD58:IgG1 fusion protein (Alefacept) approved for the treatment of psoriasis has been shown to have agonistic properties^27^ that might be useful for MS treatment. Moreover, the rs2300474 variant and rs1335532, both in high LD with rs12025416, have been associated with the autoimmune diseases neuromyelitis optica^28,29^ and primary biliary cholangitis^30^. Lastly, the rs12025416 risk allele found in this study (A/T allele) was found to have higher IL-6 and TNF-alpha macrophage response to *Candida* exposure^31^, suggesting that another possible mechanism for increased MS risk may be related to a pro-inflammatory state induced by a stronger TNF-alpha response, possibly through decreased CD58 stimulation on macrophages leading them to take on a pro-inflammatory state, consistent with other MS susceptibility variants showing an enrichment in the TNF-alpha pathway^15,16^.

Surface expression of FCRL3, an immunoglobulin receptor, on B cells has been associated with increased risk for several autoimmune diseases, including systemic lupus erythematosus, autoimmune thyroid disease, Graves’ disease and rheumatoid arthritis^32–39^, though incongruously, the same allele (which is in high LD with the risk allele of the rs6681271 variant) associated with increased FCRL3 expression and risk for these diseases was found to be associated with protection from MS^40,41^, and the association with SLE was not present in African-American, Korean or European-American groups^36,42–44^. Nevertheless, FCRL3 stimulation via secretory IgA has recently been shown to promote a pro-inflammatory phenotype, in part by inhibiting the suppressive effects of regulatory T cells^45^, as previously shown^46^, and the risk allele of the rs6681271 variant has been shown to increase FCRL3 expression^39,47^, consistent with an increased MS risk (assuming the increased expression promotes increased total FCRL3 stimulation on regulatory T cells). Similarly, expression of its homolog FCRL1 was higher in patients with MS^48^. Thus, FCRL3 (or secretory IgA) inhibition represents one potential strategy for an MS therapeutic.

In summary, two variants involved in regulation of immune responses predict a 1.44-fold increased risk of MS in African-Americans with two copies of European ancestry compared to baseline-risk African-Americans. It is likely that other genetic and environmental effects have major impacts on incidence in people of both ancestries, and future work is necessary to determine how these numerous factors interact to lead to the variable prevalence rates of MS observed in different populations today.

## Materials and methods

### Data sets

All individuals participating in the study provided full informed consent, and all experimental assays were performed in accordance with the relevant guidelines and regulations as determined by ethical review from the UCSF Human Research Protection Program Institutional Review Board (IRB) (10-05039) Protocol Title: Genetic and non-genetic risk factors for MS. We used data from samples genotyped on a specially designed Multiple Sclerosis SNP array (MS Chip), which included SNPs from the Illumina HumanExome BeadChip^49^, ancestry informative markers (AIMS), GWAS catalog SNPs for MS, and additional SNPs near and on the centromere of chromosome 1. We excluded individuals based on low call rate (average of < 98%), discrepancies between reported sex and genetically inferred sex, high autosomal heterozygosity (> 3.5 standard deviations above the mean), principal components analysis (PCA) outliers, and high relatedness with other samples (PLINK PI_HAT > 0.2)^50,51^. We excluded SNPs based on low call rate (< 98%), discordance with plate controls, Hardy–Weinberg disequilibrium (p < 0.00001), and differential missingness between cases and controls (p < 0.001). After QC exclusions, there were 2460 individuals genotyped at 300,287 SNPs (from a start of 2630 individuals and 321,105 SNPs) available for analysis (1305 cases and 1155 controls). We also used summary statistics from MS Chip data^15^ in a set of 39,238 European samples from 7 different European populations (European ancestry individuals in Australia, Denmark, Italy, Greece, Sweden, UK, and US for a total of 20,282 cases and 18,956 controls).

### Admixture mapping

An AIM panel was constructed based on information from Tandon et al*.*^52^ as well as additional SNPs near the centromere of chromosome 1 with high allele frequency differentiation (> 50% difference in minor allele frequency) between YRI and CEU populations in the 1000 Genomes Phase 3 data^53^. SNPs were LD-pruned by removing one SNP per pair with r^2^ > 0.2 in the CEU and YRI populations and one SNP per pair with genetic distance < 0.005 Morgans. SNPs with estimated frequencies that do not match the parental frequencies were removed as suggested by Patterson et al.^1^ ANCESTRYMAP was used to analyze the data with LOD score for association defined as the ratio of the likelihood of the data under a disease model divided by the likelihood of the data under no disease model. Local and global estimates of ancestry were also obtained with the ANCESTRYMAP software. For most runs, ANCESTRYMAP was set to have risk = 1.5 and the following parameters: splittau: YES, numburn: 100, numiters: 200, emitter: 30, cleaninit: YES, resitter: 5, with the rest of the parameters set to default. The risk was set to different numbers in the calculation of a 95% confidence interval as described below. Empirical p-values were determined by permuting Case and Control status and running ANCESTRYMAP for each different subset (all cases and all controls, 2007 cases and all controls, non-2007 cases and all controls, and old 2007 cases and controls from the previous genotyping platform) 1000 times and determining the maximum LOD score across all chromosomes, then comparing these scores with the maximum LOD score of the different data subsets. p-values were calculated as the proportion of times (out of 1000) that the permuted run produced a maximum LOD score greater than the maximum LOD score of that data subset. In these analyses there is no multiple hypothesis testing so a statistical significance of p < 0.05 can be used.

### Tests for statistical significance of alleles

We ran logistic regressions in R of genotype on case–control status as the main tests of association while controlling for local and genome-wide estimates of European ancestry (calculated within the ANCESTRYMAP software during the admixture mapping runs for these samples) as covariates. For these analyses we used the command glm(CaseControl ~ Genotype + GlobalEuropeanAncestry + LocalEuropeanAncestry, family = binomial(logit)). We also ran the reverse test regressing local European ancestry onto case–control status and controlling for the genotypes of the top variants from the above association as a covariate (glm(CaseControl ~ LocalEuropeanAncestry + GlobalEuropeanAncestry + TopVariant, family = binomial(logit))). For this second analysis we calculated local European ancestry using ANCESTRYMAP using interpolated scores at 1 cM intervals across the admixture association peak. To calculate the 95% confidence intervals, we ran ANCESTRYMAP with the different data subsets of Table 1 at risk models from 0.50 to 2.0 in intervals of 0.01 and found the top LOD score on Chromosome 1 for each model. We then obtained the raw scores as 10 to the power of the (LOD score), normalized the scores to sum to 1 within each data subset, and calculated the confidence intervals as the intervals that contain 95% of the normalized score, with the outer edges each summing to 2.5% (this assumes a prior with equal weight on each of the risk models). LD between different variants (r^2^) was calculated using PLINK version 1.90^50,51^ using the –ld command. Power analyses of SNP association in the African-American data were calculated using the calculator provided at: https://github.com/kaustubhad/gwas-power (accessed July 26, 2020) derived from Appendix 1 of Visscher et al.^54^. The power_beta_maf function was used with beta values varying from 0.1 to 0.4, maf (minor allele frequency) varying from 0.05 to 0.5, n = 899, and pval = 5E-8. The power for the rs12025416 and rs6681271 variants were calculated using odds ratios of 1.37 and 1.07, minor allele frequencies of 0.162 and 0.490, n = 899, and pval = 5E-8.

### Population attributable risk

The population attributable risk (PAR) was calculated as 1–1/(total reduced risk), where the total reduced risk was: α^2^*p^2^ + 2*α*(p)*(q) + q^2^, where p is the frequency of the variant, q = 1 − p, and α is the odds ratio of the variant’s effect in the population. For two variants, the total reduced risk was calculated as: (α_1_^2^*p_1_^2^*α_2_^2^*p_2_^2^) + (2*α_1_^2^*p_1_^2^*α_2_*p_2_*q_2_) + (α_1_^2^*p_1_^2^*q_2_^2^) + (2*α_1_*p_1_*q_1_*α_2_^2^*p_2_^2^) + (4*α_1_*p_1_*q_1_*α_2_*p_2_*q_2_) + (2*α_1_*p_1_*q_1_*q_2_^2^) + (q_1_^2^*α_2_^2^*p_2_^2^) + (2*q_1_^2^*α_2_*p_2_*q_2_) + (q_1_^2^*q_2_^2^), where the subscripts indicate the variant. The risk for African-Americans was calculated assuming a model with 20% European ancestry and 80% African ancestry (global ancestry proportions estimated in ANCESTRYMAP for each individual are provided in Supplementary Table S1).

### Ethics approval and consent to participate

All individuals provided full informed consent and ethical review was provided by the UCSF Human Research Protection Program Institutional Review Board (IRB) (10-05039) Protocol Title: Genetic and non-genetic risk factors for MS.

## Supplementary information

Supplementary Legends.

Supplementary Table S1.

Supplementary Table S2.

Supplementary Table S3.

Supplementary Table S4.

Supplementary Figures.

## Data Availability

All data analyzed in this article are available upon request for MS research only per the informed consent and IRB approval (contact Jorge Oksenberg: Jorge.Oksenberg@ucsf.edu).
