# Supplementary material for: Two genetic variants explain the association of European ancestry with multiple sclerosis risk in African-Americans
Source: Sci Rep. 2020 Oct 9;10:16902. doi: 10.1038/s41598-020-74035-7 (PMC7547691; doi:10.1038/s41598-020-74035-7)
Supplement: Supplementary file 1 — Supplementary Legends. [file 41598_2020_74035_MOESM1_ESM.docx]

**Supplementary Online Tables**

**Two Genetic Variants Explain the Association of European Ancestry with Multiple Sclerosis Risk in African-Americans**

Nathan Nakatsuka^1,2,^*, Nick Patterson^3,4^, Nikolaos A. Patsopoulos^4,5,6^, Nicolas Altemose^7^, Arti Tandon^1,4^, Ashley H. Beecham^8^, Jacob L. McCauley^8,9^, Noriko Isobe^10^, Stephen Hauser^10^, Philip L. De Jager^4,11^, David A. Hafler^4,12^, Jorge R. Oksenberg^10^, David Reich^1,3,4,13,^*

^1^Department of Genetics, Harvard Medical School, New Research Building, Boston, MA 02115, USA

^2^Harvard-MIT Division of Health Sciences and Technology, Harvard Medical School, Boston, MA 02115, USA

^3^Department of Human Evolutionary Biology, Harvard University, 16 Divinity Ave., Cambridge, MA 02138, USA

^4^Broad Institute of Harvard and Massachusetts Institute of Technology, Cambridge, MA 02141, USA

^5^Systems Biology and Computer Science Program, Ann Romney Center for Neurological Diseases, Department of Neurology, Brigham & Women's Hospital, Boston, MA 02115, USA

^6^Division of Genetics, Department of Medicine, Brigham & Women’s Hospital, Harvard Medical School, Boston, MA 02115, USA

^7^Department of Bioengineering, University of California Berkeley, San Francisco, Berkeley, CA 94720, USA

^8^John P. Hussman Institute for Human Genomics, Miller School of Medicine, University of Miami, Miami, FL 33136, USA

^9^Dr. John T. Macdonald Foundation Department of Human Genetics, Miller School of Medicine, University of Miami, Miami, FL 33136, USA

^10^Department of Neurology, University of California San Francisco School of Medicine, San Francisco, CA 94158, USA

^11^Center for Translational & Computational Neuroimmunology, Department of Neurology, Columbia University Irving Medical Center, New York, NY 10032, USA

^12^Departments of Neurology and Immunobiology, Yale School of Medicine, New Haven, CT 06520, USA

^13^Howard Hughes Medical Institute, Harvard Medical School, Boston, MA 02115, USA

*Corresponding authors: Nathan Nakatsuka (nathan_nakatsuka@hms.harvard.edu) and David Reich (reich@genetics.med.harvard.edu)

**Supplementary Online Tables:**

**Online Table 1. Sample Details.** Details about each of the individuals in this study and statistical testing comparing the 2007 cases to the non-2007 cases and the controls.

**Online Table 2. Details about top MS Chip variants in the admixture mapping peak.** Details about each of the most significant SNPs in the MS Chip association within the admixture mapping peak.

**Online Table 3. Details about associations for European and African-American data.** **Tab 1)** P-values and odds ratios for each SNP on chromosome 1 for the European MS Chip association. **Tab 2)** Negative log_10_ p-values for association of local ancestry with case-control status in African-American data throughout the admixture mapping peak after conditioning for genotype value of the top MS Chip variants. **Tab 3)** Negative log_10_ p-values for association of genotype with case-control status in African-American data for the top MS Chip variants. **Tab 4)** LOD scores under different risk models for the different African-American sample sets.

**Online Table 4. LD structure of the top associated variants.** LD statistics (r^2^ and D’) of the top variants found in this study in African-American data and Africans and Europeans in 1000 Genomes.
