# Supplementary material for: Two genetic variants explain the association of European ancestry with multiple sclerosis risk in African-Americans
Source: Sci Rep. 2020 Oct 9;10:16902. doi: 10.1038/s41598-020-74035-7 (PMC7547691; doi:10.1038/s41598-020-74035-7)
Supplement: Supplementary file 6 — Supplementary Figures. [file 41598_2020_74035_MOESM6_ESM.docx]

**Supplementary Material**

**Two Genetic Variants Explain the Association of European Ancestry with Multiple Sclerosis Risk in African-Americans**

Nathan Nakatsuka^1,2,^*, Nick Patterson^3,4^, Nikolaos A. Patsopoulos^4,5,6^, Nicolas Altemose^7^, Arti Tandon^1,4^, Ashley H. Beecham^8^, Jacob L. McCauley^8,9^, Noriko Isobe^10^, Stephen Hauser^10^, Philip L. De Jager^4,11^, David A. Hafler^4,12^, Jorge R. Oksenberg^10^, David Reich^1,3,4,13,^*

^1^Department of Genetics, Harvard Medical School, New Research Building, Boston, MA 02115, USA

^2^Harvard-MIT Division of Health Sciences and Technology, Harvard Medical School, Boston, MA 02115, USA

^3^Department of Human Evolutionary Biology, Harvard University, 16 Divinity Ave., Cambridge, MA 02138, USA

^4^Broad Institute of Harvard and Massachusetts Institute of Technology, Cambridge, MA 02141, USA

^5^Systems Biology and Computer Science Program, Ann Romney Center for Neurological Diseases, Department of Neurology, Brigham & Women's Hospital, Boston, MA 02115, USA

^6^Division of Genetics, Department of Medicine, Brigham & Women’s Hospital, Harvard Medical School, Boston, MA 02115, USA

^7^Department of Bioengineering, University of California Berkeley, San Francisco, Berkeley, CA 94720, USA

^8^John P. Hussman Institute for Human Genomics, Miller School of Medicine, University of Miami, Miami, FL 33136, USA

^9^Dr. John T. Macdonald Foundation Department of Human Genetics, Miller School of Medicine, University of Miami, Miami, FL 33136, USA

^10^Department of Neurology, University of California San Francisco School of Medicine, San Francisco, CA 94158, USA

^11^Center for Translational & Computational Neuroimmunology, Department of Neurology, Columbia University Irving Medical Center, New York, NY 10032, USA

^12^Departments of Neurology and Immunobiology, Yale School of Medicine, New Haven, CT 06520, USA

^13^Howard Hughes Medical Institute, Harvard Medical School, Boston, MA 02115, USA

*Corresponding authors: Nathan Nakatsuka (nathan_nakatsuka@hms.harvard.edu) and David Reich (reich@genetics.med.harvard.edu)

**Supplementary Data:**

Supplementary Data include 6 supplementary figures, 1 supplementary table, and 4 online excel tables.

**Supplementary Figures:**


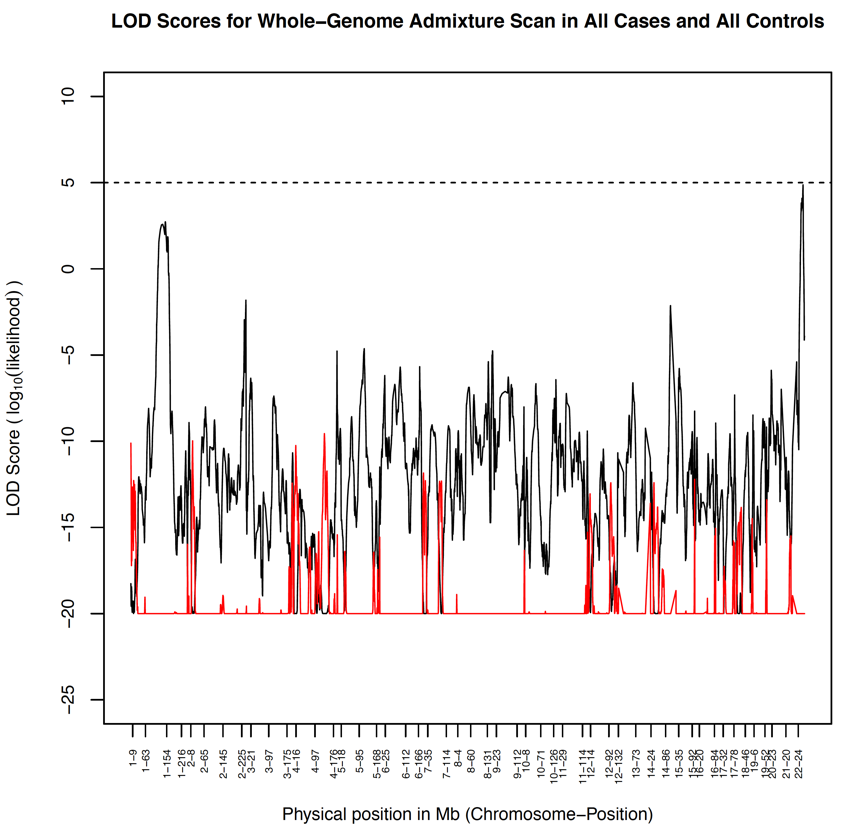


**Supplementary Figure 1. Admixture mapping LOD scores across the entire genome in an analysis of all 1,305 cases and 1,155 controls.** Results are computed by ANCESTRYMAP based on a prior on relative risk per European ancestry allele of 1.5 in black and of 0.5 in red.


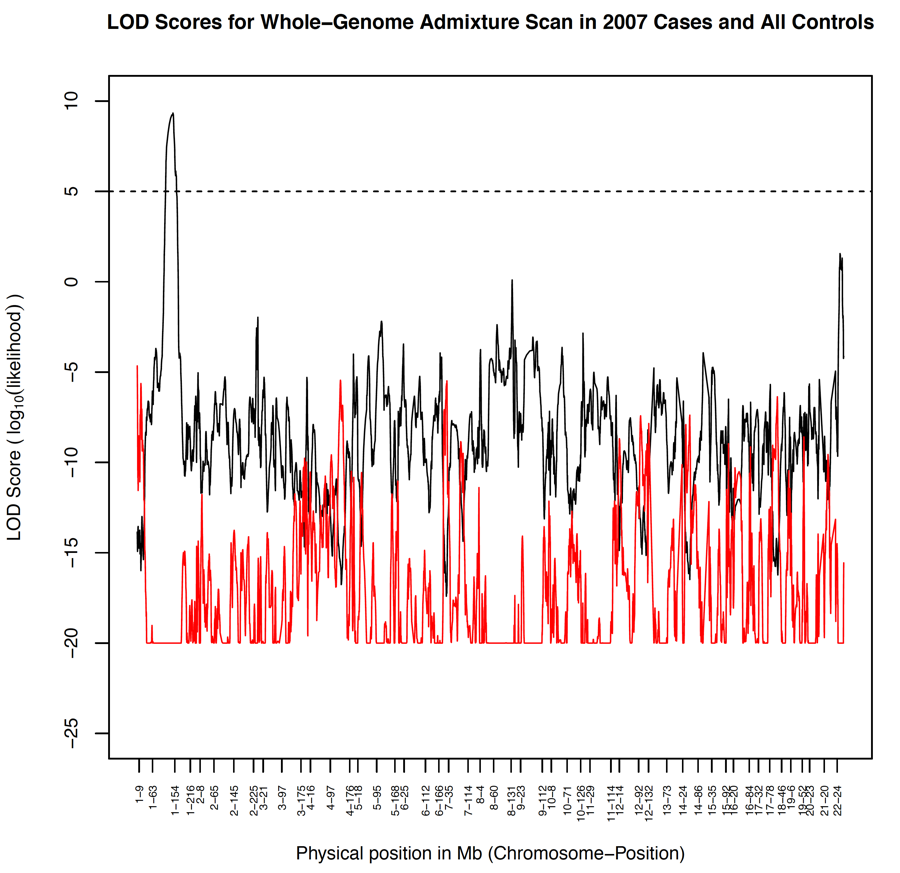


**Supplementary Figure 2. Admixture mapping LOD scores across the entire genome in an analysis of 899 cases that overlapped the 2007 follow-up of the 2005 study and all 1,155 controls.** Results are computed by ANCESTRYMAP based on a prior on relative risk per European ancestry allele of 1.5 in black and of 0.5 in red.


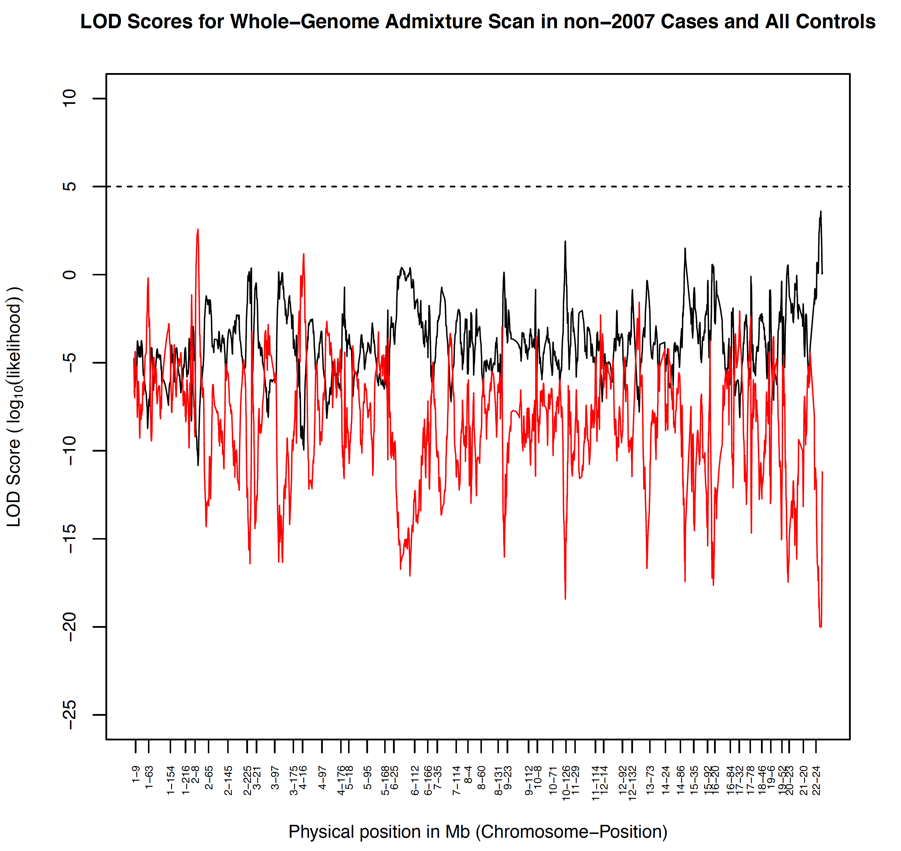


**Supplementary Figure 3. Admixture mapping LOD scores across the entire genome in an analysis of 406 cases that did not overlap the 2007 follow-up of the 2005 study and all 1,155 controls.** Results are computed by ANCESTRYMAP based on a prior on relative risk per European ancestry allele of 1.5 in black and of 0.5 in red.

| **Beta** | **Minor Allele Frequency** | | | | | | | | | |
| --- | --- | --- | --- | --- | --- | --- | --- | --- | --- | --- |
|  | **0.05** | **0.10** | **0.15** | **0.20** | **0.25** | **0.30** | **0.35** | **0.40** | **0.45** | **0.50** |
| **0.10** | *0.00* | *0.00* | *0.00* | *0.00* | *0.00* | *0.00* | *0.00* | *0.00* | *0.00* | *0.00* |
| **0.12** | *0.00* | *0.00* | *0.00* | *0.00* | *0.00* | *0.00* | *0.00* | *0.00* | *0.00* | *0.00* |
| **0.14** | *0.00* | *0.00* | *0.00* | *0.00* | *0.00* | *0.00* | *0.00* | *0.01* | *0.01* | *0.01* |
| **0.16** | *0.00* | *0.00* | *0.00* | *0.00* | *0.01* | *0.01* | *0.01* | *0.02* | *0.02* | *0.02* |
| **0.18** | *0.00* | *0.00* | *0.00* | *0.01* | *0.02* | *0.03* | *0.04* | *0.05* | *0.05* | *0.05* |
| **0.20** | *0.00* | *0.00* | *0.01* | *0.02* | *0.04* | *0.06* | *0.09* | *0.10* | *0.12* | *0.12* |
| **0.22** | *0.00* | *0.00* | *0.02* | *0.05* | *0.08* | *0.13* | *0.17* | *0.20* | *0.23* | *0.23* |
| **0.24** | *0.00* | *0.01* | *0.04* | *0.09* | *0.16* | *0.23* | *0.30* | *0.35* | *0.38* | *0.39* |
| **0.26** | *0.00* | *0.02* | *0.07* | *0.16* | *0.27* | *0.37* | *0.46* | *0.52* | *0.55* | *0.56* |
| **0.28** | *0.00* | *0.03* | *0.12* | *0.26* | *0.41* | *0.53* | *0.62* | *0.68* | *0.72* | *0.73* |
| **0.30** | *0.00* | *0.05* | *0.20* | *0.39* | *0.56* | *0.69* | *0.77* | *0.82* | *0.85* | *0.85* |
| **0.32** | *0.01* | *0.09* | *0.29* | *0.53* | *0.71* | *0.82* | *0.88* | *0.91* | *0.93* | *0.93* |
| **0.34** | *0.01* | *0.14* | *0.41* | *0.66* | *0.82* | *0.91* | *0.95* | *0.97* | *0.97* | *0.98* |
| **0.36** | *0.02* | *0.21* | *0.54* | *0.78* | *0.91* | *0.96* | *0.98* | *0.99* | *0.99* | *0.99* |
| **0.38** | *0.03* | *0.29* | *0.66* | *0.87* | *0.96* | *0.98* | *0.99* | *1.00* | *1.00* | *1.00* |
| **0.40** | *0.04* | *0.39* | *0.77* | *0.93* | *0.98* | *1.00* | *1.00* | *1.00* | *1.00* | *1.00* |

**Supplementary Table 1. Power Analyses for SNP Association in the African-American Cohort.** The power for SNP association in the cohort of 899 African-American cases was calculated (see Methods) under different minor allele frequencies (columns) and different effect sizes (rows), Beta (which is ln(odds ratio)) with a p-value threshold of 5*10^-8^. The power is 0.32 for an effect size with an odds ratio of 1.37 (Beta=0.315) and minor allele frequency of 0.162 (the values of the rs12025416 variant) and 0.00 for an effect size with an odds ratio of 1.07 (Beta=0.068) and minor allele frequency of 0.490 (the values of the rs6681271 variant).


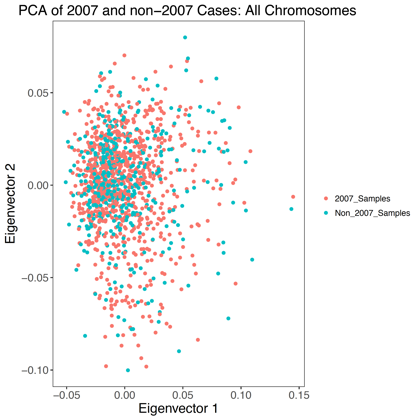

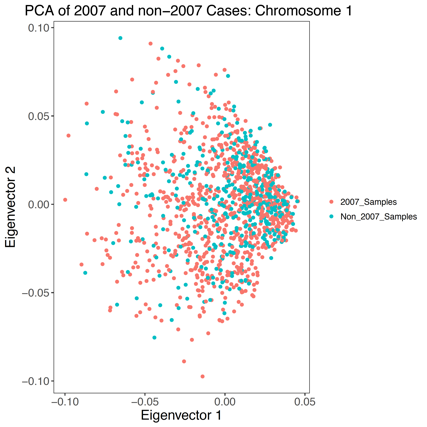

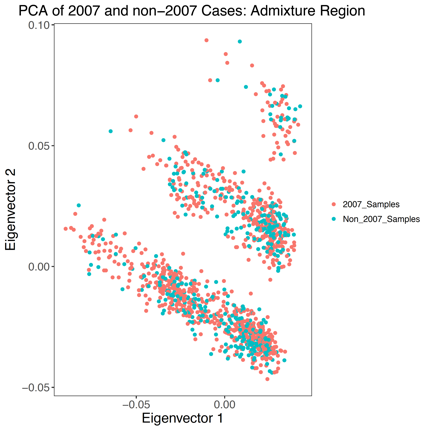


C

A

B

**Supplementary Figure 4. Principal Components Analysis (PCA) comparing 2007 and non-2007 cases. (A)** Analysis with all chromosomes. **(B)** Analysis with only Chromosome 1. **(C)** Analysis with only the region of the admixture peak near the centromere of Chromosome 1. The 2007 cases were the 899 individuals from the 2007 study that overlapped with the cases of the new cohort, while the non-2007 cases were the 406 cases that did not overlap.


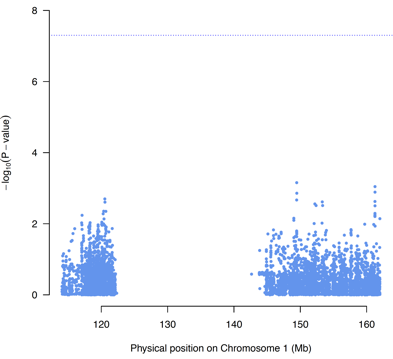

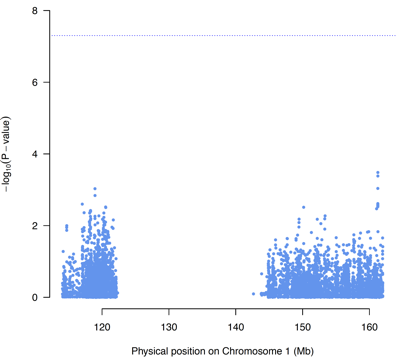


E

A

C

D

B


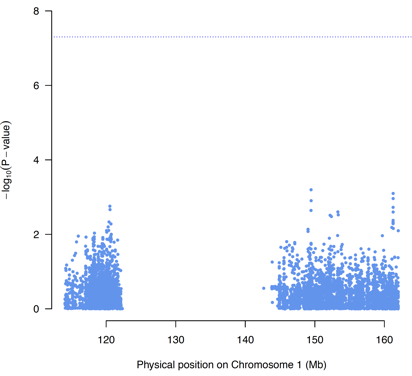

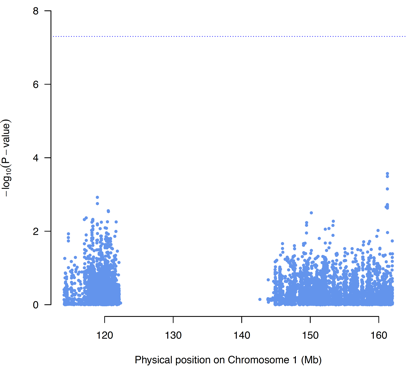


F


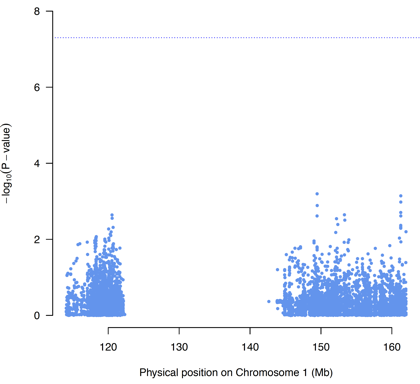

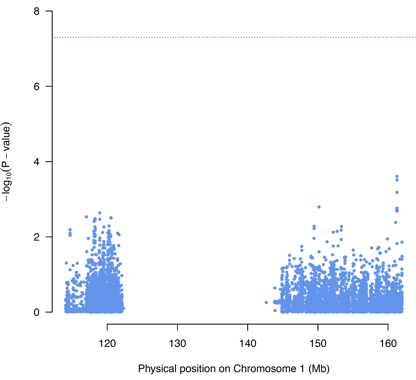


**Supplementary Figure 5. MS Chip Association in African-American data. (A)** Association of genotype on case-control status in the admixture mapping peak (114-162 Mb on chromosome 1 in hg19 genome coordinates) with only conditioning on global and local European ancestry using all cases and all controls in the African-American data. **(B)** Association with only conditioning on global and local European ancestry using the 2007 cases and all controls. **(C)** Association after conditioning on global and local European ancestry and the top 7 tag SNPs in the region most highly associated with MS in the European study using all cases and all controls. **(D)** Association after conditioning on global and local European ancestry and the top 7 tag SNPs in the region most highly associated with MS in the European study using 2007 cases and all controls. **(E)** Association after conditioning on global and local European ancestry and the top 2 SNPs in the region most highly associated with MS in the African-American data from this study using all cases and all controls. **(F)** Association after conditioning on global and local European ancestry and the top 2 SNPs in the region most highly associated with MS in the African-American data from this study using 2007 cases and all controls. Dashed line indicates significance threshold at p=5*10^-8^.

A

B


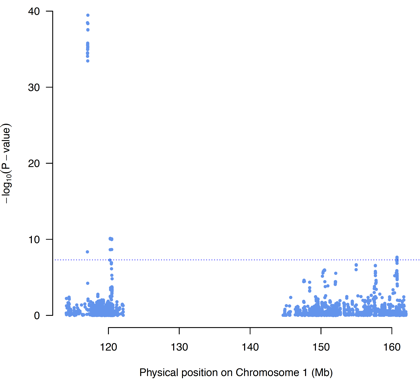

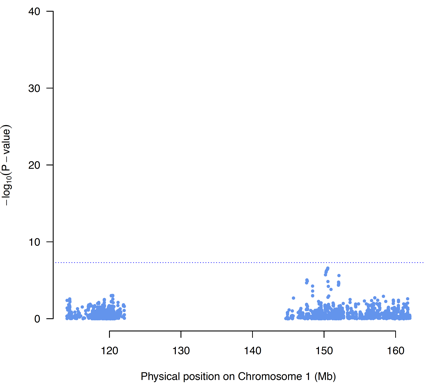

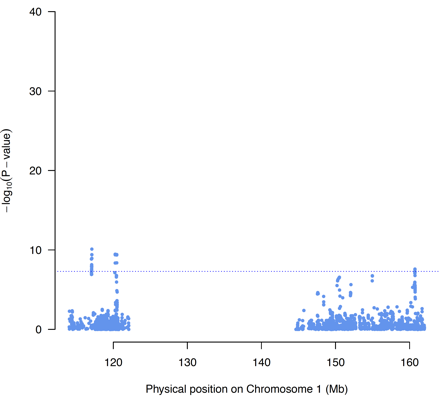


C

**Supplementary Figure 6. MS Chip Association in 39,238 Europeans. (A)** Association in the admixture mapping peak (114-162 Mb on chromosome 1 in hg19 genome coordinates) without any conditioning. **(B)** Association after conditioning with the top 7 tag SNPs in the region most highly associated with MS in the European study. **(C)** Association after conditioning with the top 2 SNPs in the region most highly associated with MS in the African-American data from this study. Dashed line indicates significance threshold at p=5*10^-8^.
